# State of the Art of Materials Science and Engineering in Italy

**DOI:** 10.3390/ma19102055

**Published:** 2026-05-14

**Authors:** Francesco Baino

**Affiliations:** Institute of Materials Physics and Engineering, Department of Applied Science and Technology, Politecnico di Torino, Corso Duca degli Abruzzi 24, 10129 Turin, Italy; francesco.baino@polito.it

Materials Science and Engineering in Italy represents a long-lasting and interdisciplinary field in which chemistry, physics, and engineering come together to produce studies and scientific results that are considered among the best in the world. This can be attributed to several factors, including the excellence of Italian academic and industrial research, as well as the outstanding capacity of Italian companies to turn innovation into valuable products. Several research groups from very different fields often collaborate in the design of materials and components, as well as in the development of processing technologies to obtain innovative products with outstanding, new, and smart properties.

This Special Issue (SI), “State of the Art of Materials Science and Engineering in Italy”, presents recent theoretical and experimental results in a range of fields, including the physics of materials, biomaterials, waste management, and metallurgy. This Editorial summarizes the seven publications (six research articles and one review paper) included in this SI. Specifically, three contributions focus on biomaterials and biomedical applications of materials, two papers address waste management, and the remaining two articles explore the fields of magnetic materials and materials for the food industry, respectively.

In contribution 1, Berni et al. investigated the effect of contrast agents on the mechanical properties of articular cartilage; this study is of great interest in the broad field of diagnosis of osteochondral pathologies [1,2].

Controlled drug delivery remains a major topic of research at the interface of biomaterials, pharmaceutics, bioengineering, and biochemistry [3]. In this regard, contribution 2 by Mazzinelli et al. explored the release of dexamethasone and clobetasol propionate from poly(lactic acid) fibers in a cellular culture system containing fibroblasts.

The development of advanced nanomaterials with antibacterial properties [4,5] aims to tackle the challenge of bacterial resistance, which is currently a key research topic. In this context, Morante et al. (contribution 3) modified silver nanoparticles and commercial BaTiO_3_ nanoparticles to obtain novel nanocomposites through sonicated sol–gel TiO_2_ synthesis and the photodeposition of Ag nanoparticles.

In the field of waste management, which is crucial for limiting the overexploitation of natural resources and reducing landfilling [6,7], Castagna et al. presented a valuable review paper (contribution 4) discussing the main extraction, processing, and bioconversion strategies for valorizing agricultural by-products.

Within the field of waste management, with particular focus on inorganic materials, Elsayed et al. (contribution 5) proposed new approaches for recycling pharmaceutical glass vials by combining alkali activation of glass particles with binder jetting printing, which are two interesting strategies that have been extensively studied by the authors at the University of Padua [8].

In the field of basic science, a very comprehensive multi-physical characterization was carried out by Halder et al. (contribution 6) to elucidate the non-magnetic behavior of some special types of iridates, which have significant potential in electronic and magnetic applications [9].

Finally, with regard to the use of metals in food packaging [10], contribution 7 by Rossi et al. reported approaches aimed at optimizing the durability and corrosion resistance of stainless steel used in the food industry, especially in relation to surface treatments and exposure to biocidal additives.

Collectively, these contributions suggest that materials science and engineering research in Italy are evolving significantly, especially in the biomedical field and agri-food sector, which are also of great interest to industries and other non-academic stakeholders.
